# Lifetime rates and types of subsequent child protection system contact following a first report of neglect: An age-stratified analysis

**DOI:** 10.1371/journal.pone.0283534

**Published:** 2023-04-12

**Authors:** Lindsey Palmer, Sarah Font, Rebecca Rebbe, Emily Putnam-Hornstein

**Affiliations:** 1 Human Development and Family Studies, Pennsylvania State University, University Park, PA, United States of America; 2 Department of Sociology and Criminology and School of Public Policy, Pennsylvania State University, University Park, PA, United States of America; 3 School of Social Work, University of North Carolina at Chapel Hill, Chapel Hill, NC, United States of America; Hiroshima International University: Hiroshima Kokusai Daigaku, JAPAN

## Abstract

An estimated 1 in 3 U.S. children will be the subject of a child protective services (CPS) investigation during their lifetime, typically for allegations of neglect. Whether and how an initial report of neglect is addressed may place children on divergent trajectories for safety and stability throughout childhood. The purpose of this study is to track subsequent CPS contact among children born in California in 2000 who were first investigated by CPS for neglect allegations alone (no co-occurring abuse) and not permanently separated from their families of origin (i.e., not removed or reunified if removed). We estimated the rates of subsequent CPS referrals, substantiated maltreatment, placement in foster care, and allegations of physical and sexual abuse by age 18. We assessed how rates of subsequent contact varied by initial CPS response and age at first investigation. Supplemental analyses disaggregated data by race and ethnicity. Results indicate that 64% of children initially investigated for neglect alone were re-referred to CPS by age 18 and 16% experienced a subsequent removal; however, these estimates varied greatly by age. Four out of five (79% to 83%) of children initially investigated as infants had one or more subsequent CPS referrals during childhood. Children were not only re-referred for allegations of neglect; more than half of children re-referred were reported for allegations of physical or sexual abuse, indicating that abuse risk was either missed during the initial CPS investigation or escalated afterward. The failure to address maltreatment risks when children first present to the system is a complex problem with no easy solution. Our findings document that a majority of children initially referred for neglect experience future CPS involvement, often for allegations of physical or sexual abuse.

## Introduction

By age 18, it is estimated that 26% to 37% of U.S. children will be the subject of a child protective services (CPS) investigation [1], typically due to neglect allegations. Neglect generally refers to a lack of minimally adequate care (failure to provide food, clothing, housing, medical care, or supervision) [2] that results in actual or imminent threat of harm to a child [3]. Neglect has well-established adverse impacts on child health and development [4,5].

Young children, who are entirely dependent on their caregivers to meet their physical and emotional needs, are especially vulnerable to the lifelong consequences of neglect. A majority of the children who encounter CPS are first reported in early childhood, a period when risk of victimization and death is highest [1,6]. Yet comparatively few children are substantiated (officially counted) as victims of maltreatment or enter foster care [7,8], especially following their first report to CPS [9], and many who enter foster care return home within a year [10]. Because parental conditions that lead to neglect are often chronic in nature and can be antecedent to physical and sexual abuse [9,11], whether and how an initial report of neglect is addressed may place children on divergent trajectories for safety and stability throughout childhood. Estimated rates of recurrence—new allegations of child maltreatment—following a prior neglect investigation range from 2% to 66%, depending on the sample or population, measure of subsequent maltreatment, and follow-up period [12]. Although studies have indicated that placement in foster care reduces subsequent maltreatment [13] and deaths [14] in the near term for children reported to CPS, studies rarely disaggregated recurrence rates based on the initial maltreatment allegation and CPS response or follow children for the duration of childhood. To our knowledge, no large-scale U.S. studies have examined, for example, how often children remaining at home after a neglect investigation are subsequently reported for sexual abuse or placed in foster care due to new allegations.

In the current study, we tracked multiple forms of subsequent CPS contact among children born in California in 2000 who were first investigated by CPS for neglect allegations alone (no co-occurring abuse allegations) and not permanently separated from their families of origin as a consequence of that investigation. Specifically, we addressed two aims: (a) to estimate the rates of subsequent referral, substantiated maltreatment, placement in foster care, and allegations of physical and sexual abuse by age 18; and (b) to assess how rates of subsequent CPS contact varied based on the outcome of the initial CPS neglect investigation and age. In supplemental analyses, we also disaggregated rates by race and ethnicity.

## Method

Data were derived from California’s Child Welfare Services/Case Management System in accordance with a data-sharing agreement with the California Department of Social Services. The analytic cohort featured all children born in California in 2000 who were the subject of at least one CPS investigation by age 18 and whose first CPS investigation was initiated based on allegations of neglect alone, without any co-reported abuse allegations. Vital birth records from the California Department of Public Health were used to identify the 2000 birth cohort to ensure observation since birth. Children whose first investigation resulted in removal but not reunified were excluded from this analysis (*n* = 2,137; representing <1% of the overall birth cohort and 4% of all children with a first investigation for neglect only). The final cohort was 43,419 children who were not permanently separated from their family of origin after an initial investigation of neglect. Records available to the research team were stripped of all direct identifiers and anonymized prior to analysis. Analyses were approved by the California Committee for the Protection of Human Subjects and met criteria for a waiver of informed consent.

### Outcome measures

The primary outcome of interest was recurrent CPS contact, measured by level of subsequent contact and type of alleged re-maltreatment. Both measures pertain only to referrals initiated after the closure date of the child’s first investigation or the end of their first placement episode if a child was removed following the initial investigation. Level of subsequent contact included a follow-up referral (regardless of investigation), substantiated allegation, and removal episode (foster care placement). For most analyses, these were categorized based on the highest level of subsequent contact after the first investigation and before age 18: (a) none, (b) uninvestigated or unsubstantiated allegation of maltreatment, (c) substantiated maltreatment allegation without removal, and (d) removal episode. The second outcome was a subsequent allegation of abuse. We measured this outcome using two non-mutually exclusive indicators: referral for alleged physical abuse and referral for alleged sexual abuse. We also defined a summary indicator of any subsequent abuse allegation. A subsequent abuse allegation was indicated if the child received any new referral for allegations of physical or sexual abuse.

### Explanatory variables

The primary explanatory variable of interest was the CPS response to the child’s first alleged neglect investigation. Children were categorized into one of three groups: children with neglect allegations that were unsubstantiated and where the child was not removed from the home (U-NR); children with allegations that were substantiated and where the child was not removed from the home (S-NR); and children who were removed to foster care and later reunified (RR). As noted previously, children removed and not reunified following their first neglect investigation (*n* = 2,137) were excluded from subsequent analyses. Additional variables of interest were age at initial investigation (infant [0–12 months], toddler [1–3 years], school-aged child [4–12 years], adolescent [13–17 years]) and race and ethnicity (White, Black, Hispanic, Asian or Pacific Islander, Native American). Children were coded as Hispanic based on the presence of a Hispanic origins indicator and could have been of any race. Age breakdowns were selected based on an overrepresentation of infants and toddlers receiving CPS referrals and interventions [1].

### Analysis

We first calculate lifetime rates of subsequent contact by age group and CPS response to the initial investigation: percent of each age-by-initial response subgroup with any subsequent CPS referral, substantiation, and removal, and any subsequent allegation of physical or sexual abuse. Given substantial differences in the initial response by age (see Table 1) and that older children have fewer years at risk of recurrent CPS contact before reaching adulthood, all results were disaggregated by age group. Because few children had a first investigation for neglect alone as a teenager, our primary analyses focused on the largest age groups only (infants, toddlers, and school-aged children). Although the primary goal of the study is to provide detailed prevalence estimates of CPS recurrence, we also produce stratified cumulative hazard graphs for each outcome and descriptive statistics on re-referral frequency and timing to clarify group differences. Cumulative hazard graphs included all age groups in order to examine whether risk of recurrence was related to the time exposed across all ages, however, given the small numbers of children reported for the first-time during adolescence, those results should be interpreted with caution.

**Table 1:** Descriptive characteristics of children born in 2000 whose first investigation was for neglect alone, overall and by age at first investigation.

	Overall			Infant			Toddler		School age		Adolescent	
*n*	43,419			10,743			12,687		15,851		4,138	
%	100			25			29		37		10	
	*n*	%	*n*		%	*n*		%	*n*	%	*n*	%
Outcome of first investigation												
Unsubstantiated	31,857	73	6,418		60	9,288		73	12,516	79	3,635	88
Substantiated and no removal	9,060	21	3,063		29	2,739		22	2,819	18	439	11
Removed and reunified	2,502	6	1,262		12	660		5	516	3	64	2
Race and ethnicity												
White	11,492	26	3,211		30	3,444		27	3,906	25	931	22
Black	5,884	14	2,138		20	1,727		14	1,683	11	336	8
Hispanic	21,238	49	4,471		42	6,160		49	8,455	53	2,152	52
Asian or Pacific Islander	1,507	3	283		3	406		3	607	4	211	5
Native American	324	1	138		1	97		1	71	0	18	0
Subsequent CPS involvement												
Referral	27,787	64	8,483		79	9,230		73	8,840	56	1,079	26
Substantiated allegation	12,449	29	5,041		47	4,487		35	2,760	17	161	4
Removal	7,101	16	3,675		34	2,280		18	1,064	7	82	2
Physical abuse allegation	11,498	26	3,954		37	4,065		32	3,182	20	297	7
Sexual abuse allegation	5,981	14	2,031		19	2,125		17	1,652	10	173	4

## Results

### Cohort description

Table 1 details the demographic and CPS characteristics of the cohort of children whose initial investigation was for neglect alone, overall and by age at initial investigation. The racial and ethnic composition of the cohort was 26% White, 14% Black, 49% Hispanic, 3% Asian or Pacific Islander, 0.75% Native American, and 7% missing. One in four children had their first investigation during infancy (0–12 months), 29% as a toddler (1–3 years), 37% as a school-aged child (4–12 years) and 10% during adolescence (13–17 years). Seventy-three percent of the 43,419 children in our neglect cohort had an initial response of unsubstantiated with no removal (U-NR); 21% had an initial response of substantiated with no removal (S-NR); and 6% were removed and then reunified (RR). Again, 2,127 children who were removed and never reunified were excluded from our analysis.

### Recurrent CPS contact

**Fig 1:**
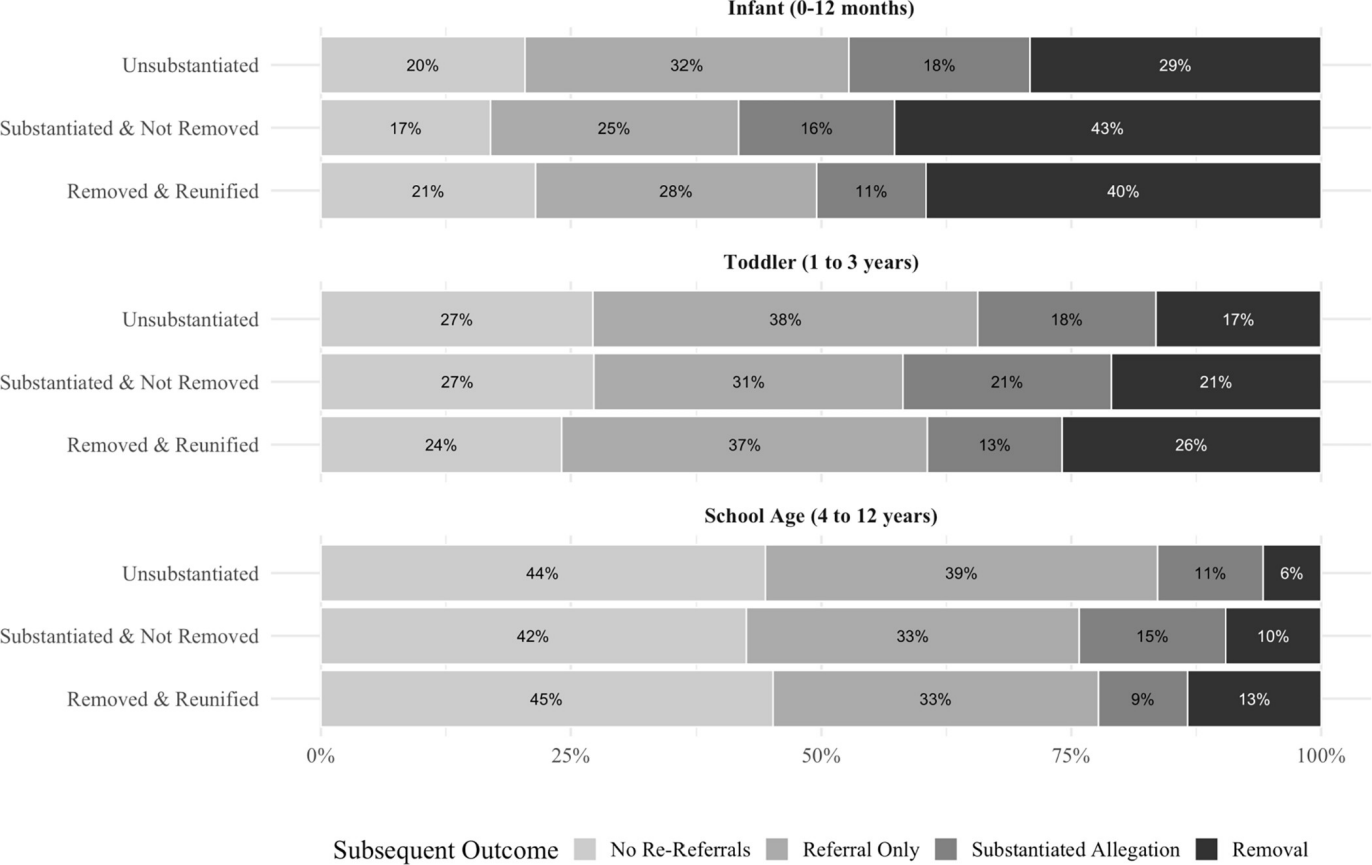
Percentage of children with subsequent CPS contact by initial response and age at first investigation.

As noted in Table 1, 64% of children whose initial CPS investigation was for neglect alone were re-referred to CPS by age 18. More than a quarter of these children (29%) had a subsequent substantiated allegation and 16% experienced a subsequent removal. These estimates varied greatly by age and, to a lesser extent, initial investigative response. Fig 1 illustrates the percentage of children with subsequent contact by initial response. Approximately 4 in 5 U-NR infants (80%), S-NR infants (83%) and RR infants (79%) had at least one subsequent CPS referral. The percentage experiencing a subsequent removal was particularly high among children first investigated as infants. S-NR and RR infants had the highest rates of subsequent removal episodes (43% and 40%, respectively), although 29% of U-NR infants also had a subsequent removal episode. Approximately 1 in 5 S-NR (21%) and U-NR (17%) toddlers experienced a subsequent removal episode, and 1 in 4 (26%) RR toddlers experienced a subsequent removal episode. Children initially investigated at ages 4 to 12 years were generally less likely to have subsequent contact across all initial response categories: 55% to 58% had at least one subsequent referral, and between 6% and 13% experienced a subsequent removal episode.

**Fig 2:**
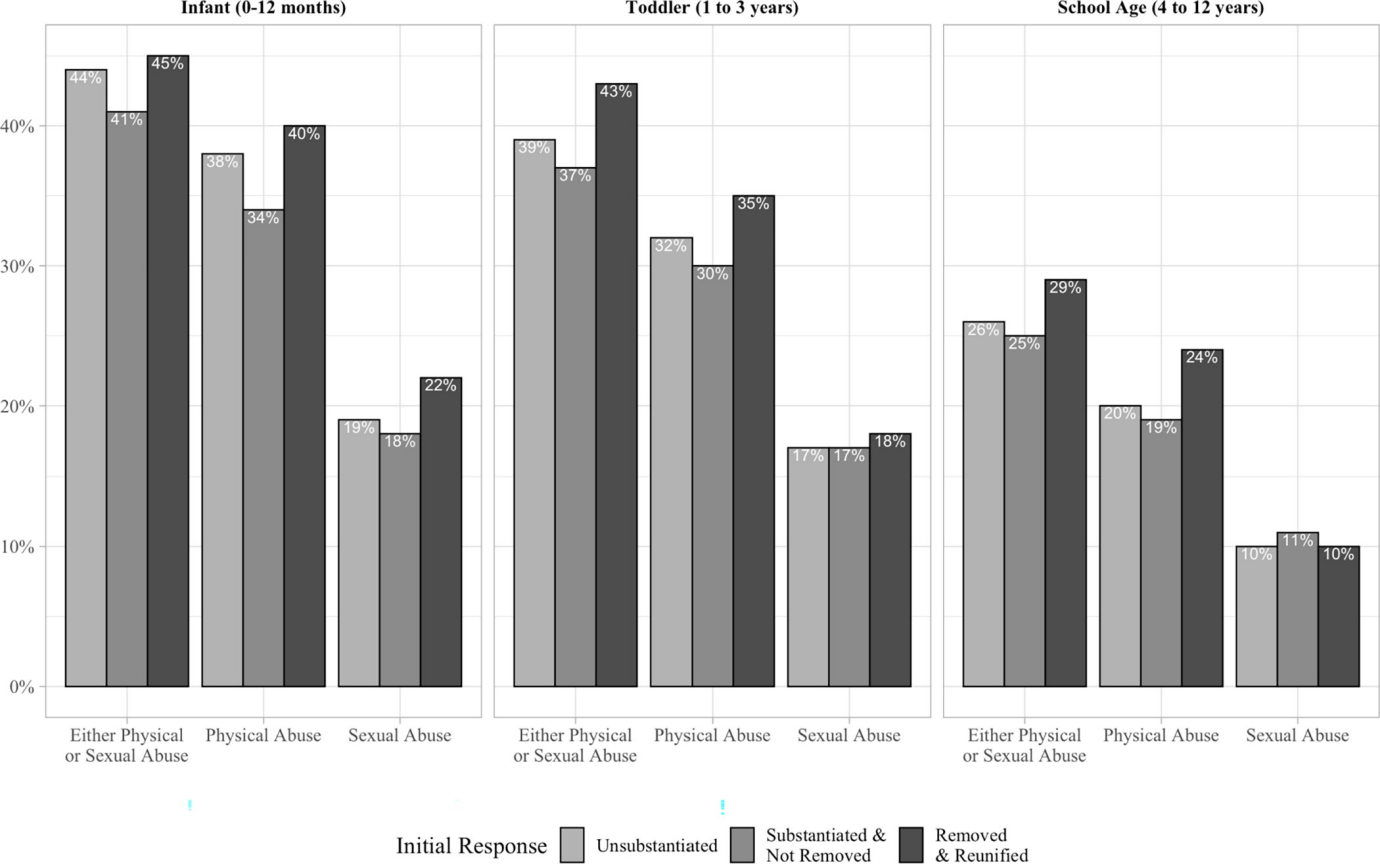
Percentage of children with subsequent CPS referrals for allegations of physical and sexual abuse by initial response and age at first investigation.

Fig 2 shows subsequent CPS referrals for allegations of physical and sexual abuse by initial response and age at first investigation. Among the full cohort of children investigated for neglect but for whom the first investigation did not lead to permanent removal from their family of origin, more than one third (33%) were subsequently referred for alleged abuse, including 14% for alleged sexual abuse and 26% for alleged physical abuse. Of those initially investigated as infants, approximately 40% were subsequently referred for alleged physical or sexual abuse across all initial response categories. Rates of subsequent abuse allegations were nearly as high (37%–43%) for children first investigated for neglect as toddlers and lower (25%–29%) among children first investigated at ages 4 to 12 years. Rates of subsequent abuse referrals—physical abuse referrals, specifically—were slightly higher for RR children than U-NR and S-NR children. For example, 40% of RR infants and 35% of RR toddlers were subsequently referred for physical abuse concerns, versus 34% and 30% of same-age S-NR children. Approximately 1 in 6 children investigated as infants or toddlers and 1 in 10 school-aged children were subsequently referred for suspected sexual abuse, with little difference by initial response.

#### Timing and number of subsequent contacts

**Fig 3:**
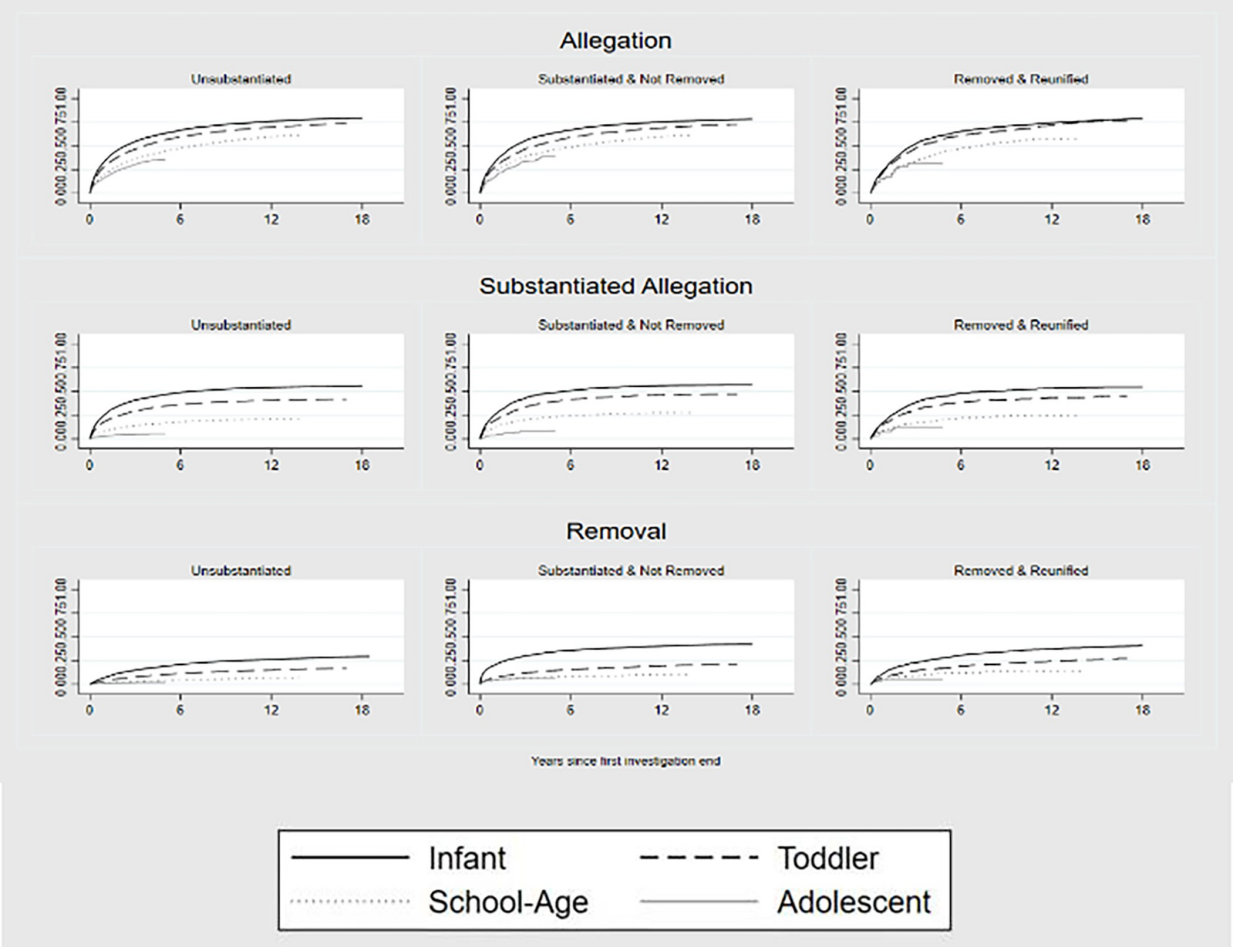
Kaplan-Meier failure curves for subsequent referral, substantiated allegation and removal, by response and age at initial investigation.

To confirm that age at initial investigation was a risk factor for subsequent CPS involvement and not a function of the time observed, we produced Kaplan-Meier cumulative failure curves for subsequent CPS referrals. Fig 3 illustrates the timing of first subsequent involvement (allegation, substantiated allegation, removal) stratified by age at and response to initial investigation and demonstrates that infants and young children experienced subsequent involvement at higher rates and more quickly compared to children initially investigated at school age or adolescence. Among the 27,787 children who had one or more subsequent CPS contacts, the median time between the closure of the initial investigation or initial placement episode, and the subsequent allegation was 18 months. Children who experienced a subsequent removal and placement into care had a median of 10 months between closure of initial investigation or placement and subsequent CPS referral.

**Fig 4:**
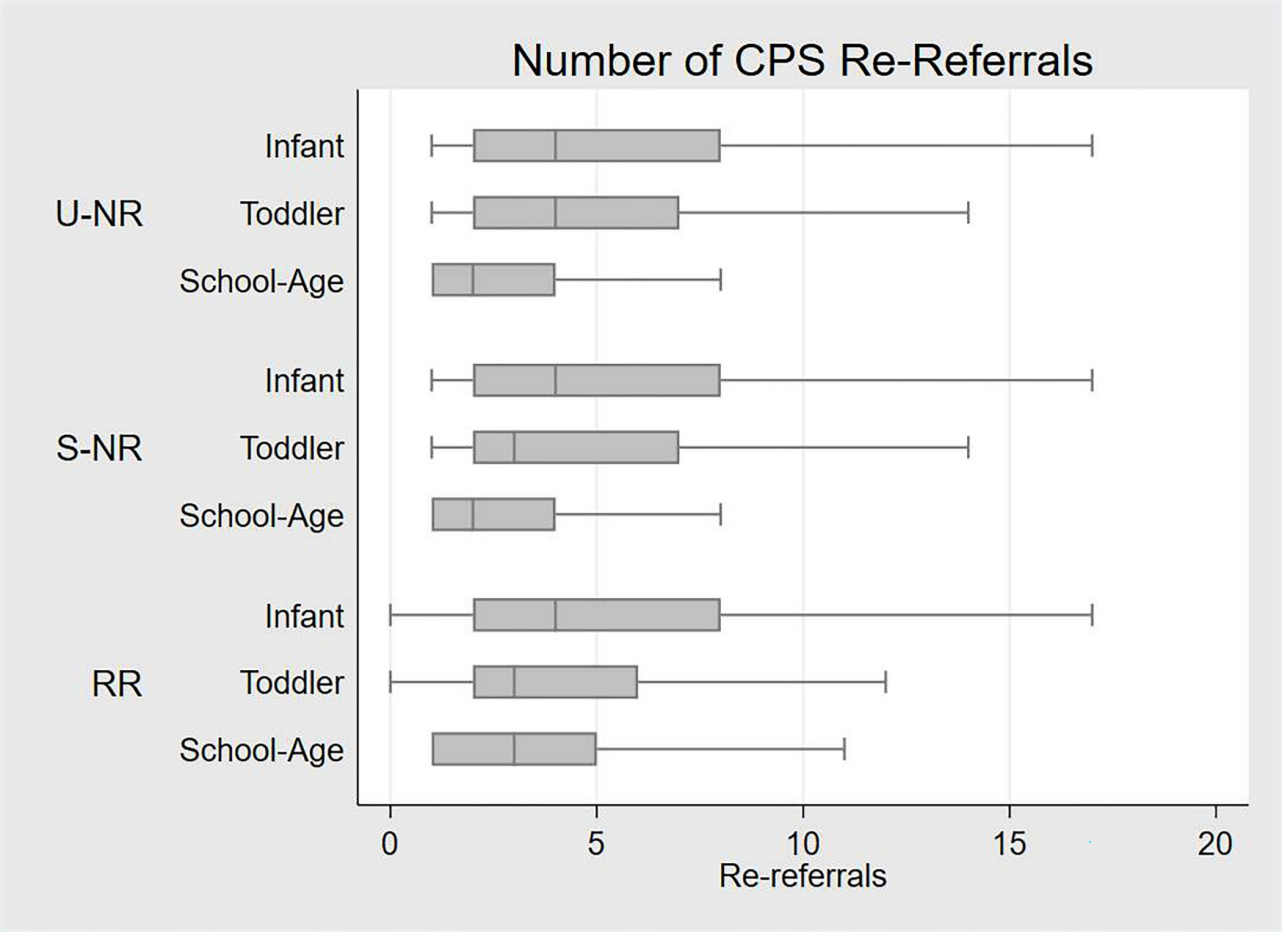
Median number of subsequent referrals following the closure of the initial investigation or placement by initial response and age at first investigation.

The prevalence and failure estimates (Figs 1–3) focus on ever or first recurrence, However, some children experience chronic or multiple recurrences of CPS contact. Children with subsequent referrals had an average (mean) of five re-referrals (median = 3; interquartile range [IQR] = 1–6); however, for children investigated as infants and later referred, the mean number of subsequent referrals was six (median = 4; IQR = 2–8; see Fig 4). Children who experienced a subsequent removal and placement into care had an average of eight CPS re-referrals prior to age 18 (median = 6; IQR = 3–10).

### Additional analyses

#### Racial differences

Supplementary analyses examined the proportion of children with subsequent contact by race and ethnicity (grouped across age and initial response), and results are shown in S1 and S2 Figs. Few observed differences emerged in subsequent removal episodes by race and ethnicity among children first investigated as infants, regardless of initial response. Among children first investigated as toddlers or older (1–12 years), Black children were more likely than White and Hispanic children to experience a subsequent removal, and racial and ethnic differences in subsequent removal were largest for S-NR children. Black children were more likely to have subsequent referrals due to allegations of physical abuse across nearly all ages and initial response categories. For most age and initial response categories, Hispanic children were less likely to have subsequent contact. In general, there were few differences across race and ethnicity for subsequent allegations of sexual abuse.

#### Surveillance bias

It is possible that subsequent referrals are merely a continuation of a flawed and overreaching surveillance system or repeated calls from the same individual, rather than a signal of ongoing risk or harm. Thus, one approach to thinking about whether rates of subsequent contact may reflect oversurveillance or reporter bias is to consider whether individuals from more than one system are reporting concerns about the same child. Individuals in one system are not privy to CPS referrals or allegations in other systems (because records are not public); thus, reporters in different systems cannot systematically respond to the label attached to a family through previous CPS contact. Furthermore, a child is more likely to be at serious risk of harm when individuals encountering the child in different settings separately identify concerns that they feel compelled to report. Our data suggest that among children who had a reporter source documented (i.e., not missing) for both the initial referral and at least one subsequent referral (67%), nearly all (94%) were re-referred by a different reporting source (e.g., initial referral by a medical provider and subsequent referral by a teacher). Relatively minor differences across race and ethnicity were observed, with White and Black children being more likely to have a greater number of different reporters than Hispanic children. Initial reports of neglect were most frequently made by police (20%), followed by medical professionals (16%), other professionals (16%), and school or daycare staff members (14%).

## Discussion

In the current study, we examined lifetime rates and levels of recurrent CPS contact among the 2000 California birth cohort of children whose first CPS investigation was due to allegations of neglect alone and who were not permanently separated from their family of origin as a result of that initial investigation. We sought to understand ongoing patterns of CPS contact to provide a baseline of future risk and highlight several findings.

First, recurrent CPS referrals are the norm for children initially investigated for neglect, especially those first investigated early in life. Nearly two thirds (64%) of children were re-referred to CPS, including more than 80% of children initially investigated as infants. Further, children who were re-referred had an average of three subsequent CPS referrals, and 25% of re-referred children had four or more subsequent referrals. Prior estimates of recurrent CPS contact vary widely due to studies using data from varying time periods and jurisdictions and with varying follow-up periods, outcome measures (re-referral, re-report, or re-victimization) and target populations and sampling frames (e.g., children reunified from foster care) [12]. Our estimates of subsequent CPS contact were higher due to the features of this study. Specifically, we were able to follow children for the entirety of their childhood (complete observation window) and identified new referrals regardless of whether they were screened in or substantiated (broader range of outcomes). In addition, we excluded children who were permanently separated from their families of origin, who may have lower rates of recurrent CPS contact. On the whole, our study confirms that children who remain with or return to their families of origin following an investigation of neglect—the vast majority of children alleged to have been neglected—experience high rates of recurrent CPS contact throughout childhood.

Second, recurrent CPS contacts appear indicative of heightened risk of maltreatment. Although CPS contact can indicate unnecessary surveillance, the patterns of recurrent involvement in this study do not appear to be consistent with frivolous or overzealous reporting. Instead many children were re-referred for maltreatment by differing reporters that resulted in a substantiated allegation or placement in foster care, and again, these risks were heavily concentrated among children initially reported as infants or toddlers. For example, more than one third of infants left at home after a first investigation of neglect entered foster care at some point in childhood and 40% of reunified infants later returned to foster care. Moreover, many of the children with subsequent contact returned to the attention of the system not–or not only–for continued neglect concerns. More than one third of children were re-referred for physical or sexual abuse, accounting for more than half of all children with subsequent referrals. Among infants, 34% to 38% of those left in the home initially and 40% of those reunified following a removal were re-referred for physical abuse and 18% to 22% were re-referred for sexual abuse. Overall, more than half of children who came back to the system were re-referred for allegations of physical or sexual abuse. These estimates are not outliers, rather, they are consistent with previous analyses finding that children initially investigated for neglect are at heightened risk of physical and sexual abuse [15,16]. Future risk of abuse allegations may indicate either an escalation of risk within their environment or maltreatment missed during the initial investigation. Given the descriptive aims of the current study, we did not attempt to adjust for differences in initial maltreatment characteristics or family contexts and thus, cannot ascertain whether the initial or subsequent CPS contacts were appropriate or necessary. However, for the subset of cases where this information was available, the vast majority of re-referrals were made by people in different systems or roles (e.g., teacher, medical professional, therapist) than the initial report, suggesting that multiple people encountering the child in varying contexts registered safety concerns. Together, these three pieces of evidence—the high rates of subsequent substantiated maltreatment and placement in foster care, high rates of subsequent concern for physical or sexual abuse, and most children being reported by multiple sources—suggest meaningful continuation of risk that is unlikely solely due to surveillance mechanisms.

Third, high rates of subsequent involvement highlight the challenges CPS workers face when examining long-term risk and raise concerns about the efficacy of available interventions. Children with substantiated and unsubstantiated neglect allegations were re-referred at similar rates, consistent with research indicating that substantiation is a poor predictor of child safety, risk, or well-being [17–19]. However, children whose initial neglect allegations were substantiated but did not lead to removal were significantly more likely than children with unsubstantiated initial investigations to have a subsequent investigation resulting in removal, again highlighting the challenges CPS faces in addressing longer-term risk or potentially indicating a reluctance to use foster care without a documented pattern of prior behavior or unresolved risk. Similarly, children removed following their initial investigation and later reunified had extremely high rates of recurrent referrals, substantiated maltreatment, and reentry to foster care.

Together, these findings raise serious questions as to the effectiveness of interventions and the level of continued support that may be needed to safely keep children with their families of origin. Interventions, especially for families with unsubstantiated allegations, are often limited to voluntary referrals for community services [20]. This study is unable to discern why recurrence and revictimization are so prevalent, but three areas warrant further consideration: identification of risk, intervening in response to risk, and the effectiveness of the response. First, CPS may underestimate the risk of future harm and thus fails to offer the range or intensity of treatment services that are needed. Though data on the treatment services provided by CPS is limited and of low-quality, it is generally reported that most investigated families receive no services or informal and short-term services [20]. Second, high-risk families may decline voluntary treatment services and CPS may be unwilling or unable to obtain a court order to mandate participation [21]. Lastly, the services provided to families may have small, null, or time-limited impacts on risk of future harm [22]. The Family First Prevention Services Act seeks to increase the use of evidence-based treatment services. However, foster care avoidance, not child safety, is the primary goal of Family First and it is not clear what, if any impacts on child safety will emerge. Nevertheless, our findings demonstrate a need to better understand whether and how families receive services following an initial investigation, and the impact of those services on system return. Systematic auditing of revictimization cases to identify errors in risk assessment and decision-making, or limitations in service effectiveness, may facilitate quality improvement efforts.

### Limitations

To our knowledge, this is the first population-level study to track recurrent CPS contact by initial CPS response from birth to age 18. Despite the strengths of the data, several limitations reinforce a need for further research and necessitate caution in interpreting findings. First, we could not discern whether families received voluntary or court-ordered services in the community or the nature and quality of such services. Although in-home services are the primary response to child neglect, we know very little about how many families involved with CPS are offered and accept these services or how impactful they are for various types of parenting concerns [20]. In short, we cannot say with certainty whether following the initial investigation, families were not offered services, declined services, or received services that were not consistently effective in reducing long-term risk [23]. Second, due to the small number of guardianship cases and concerns about the ability to track adoptees, our sample was limited to children who were not initially removed or removed but reunified. More comparative studies are needed on long-term safety and well-being outcomes following reunification, adoption, and guardianship. Third, given the variability across state systems, findings cannot be generalized to the national level unless confirmed by replication studies in other jurisdictions.

### Conclusion and implications

Young children are particularly vulnerable to neglect and constitute an outsize share of initial investigations for neglect—half of first neglect investigations involved children from infancy to 3 years old. Yet, the vast majority of initial CPS investigations of neglect are unsubstantiated (73%), and only a small minority of suspected victims enter foster care (6%), indicating light or no services for most children and families [24]. Further, children initially reported for neglect, especially early in life, are highly likely to have at least one subsequent CPS contact, frequently involving suspicion of physical or sexual abuse or necessitating subsequent intervention. Given the heightened risk of adverse outcomes associated with chronic maltreatment [25] and that it is more difficult to rebound from the effects of early trauma as children age, it is concerning to see that nearly 80% of infants investigated for neglect are re-referred to CPS and more than one third are re-referred for concerns severe enough to warrant foster care placement. The failure to address maltreatment risks when children first present to the system is a complex problem. A persistent tension exists among three issues: the limited window of sensitive periods for early childhood development [26], the lack of sustained efficacy for interventions, and the “last resort” nature of foster care and adoption [27]. Further complicating these issues in the U.S. context is a weak child maltreatment prevention infrastructure [28], coupled with the reality that most at-risk families decline voluntary services [21]. These longstanding challenges have no easy solutions. This study highlights that for many children, current efforts to address neglect are inadequate—in intensity, scope, duration, or efficacy—to ameliorate risk.

## Supporting information

S1 Fig Percentage of children with subsequent contact by initial response, race and ethnicity, and age at first investigation. (TIF)

S2 Fig Percentage of children with subsequent referrals of physical and sexual abuse by initial response, race and ethnicity, and age at first investigation. (TIF)
