# Supplementary figures and images for: Lifetime rates and types of subsequent child protection system contact following a first report of neglect: An age-stratified analysis
Source: PLoS One. 2023 Apr 12;18(4):e0283534. doi: 10.1371/journal.pone.0283534 (PMC10096237; doi:10.1371/journal.pone.0283534)

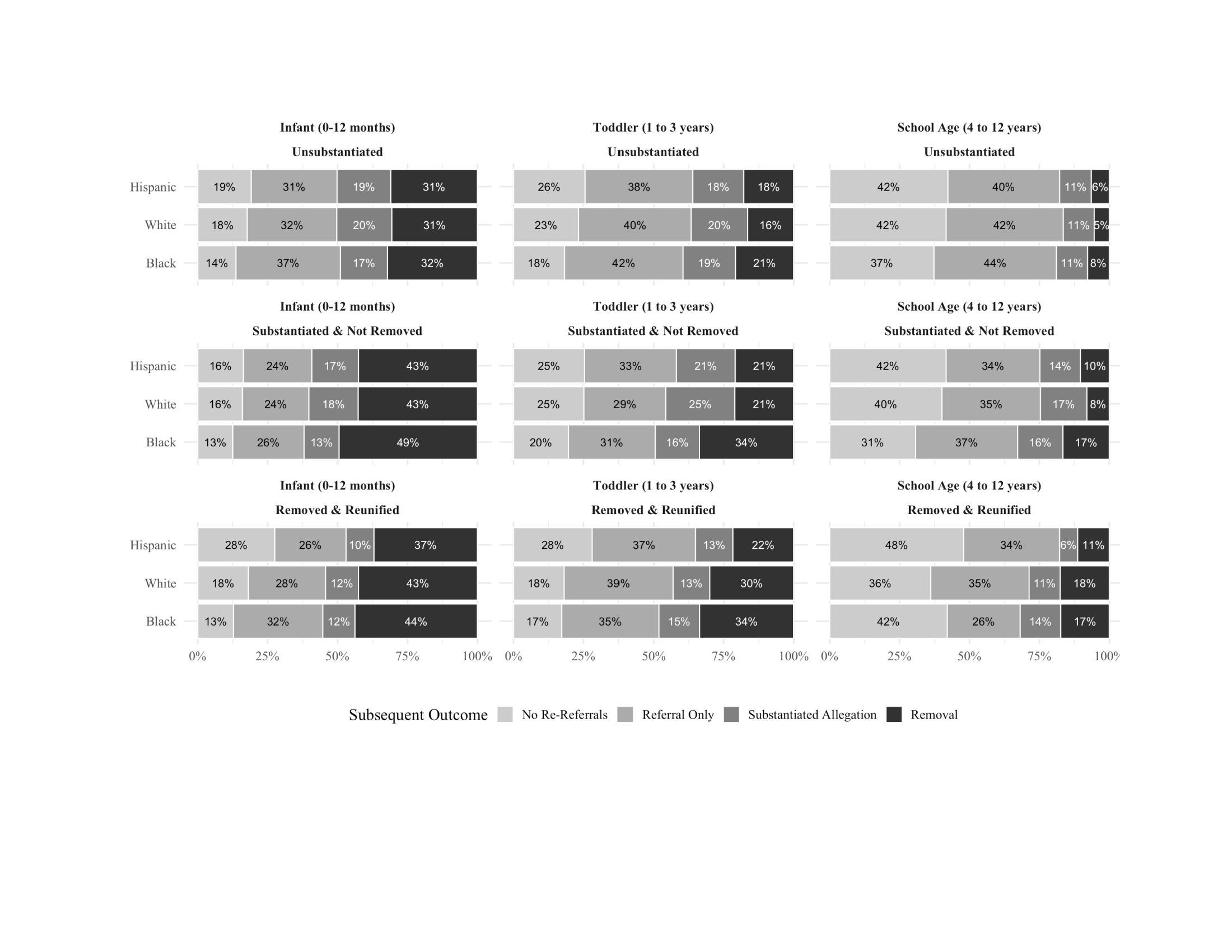

Supplement: S1 Fig — (TIF) [file pone.0283534.s001.tif]

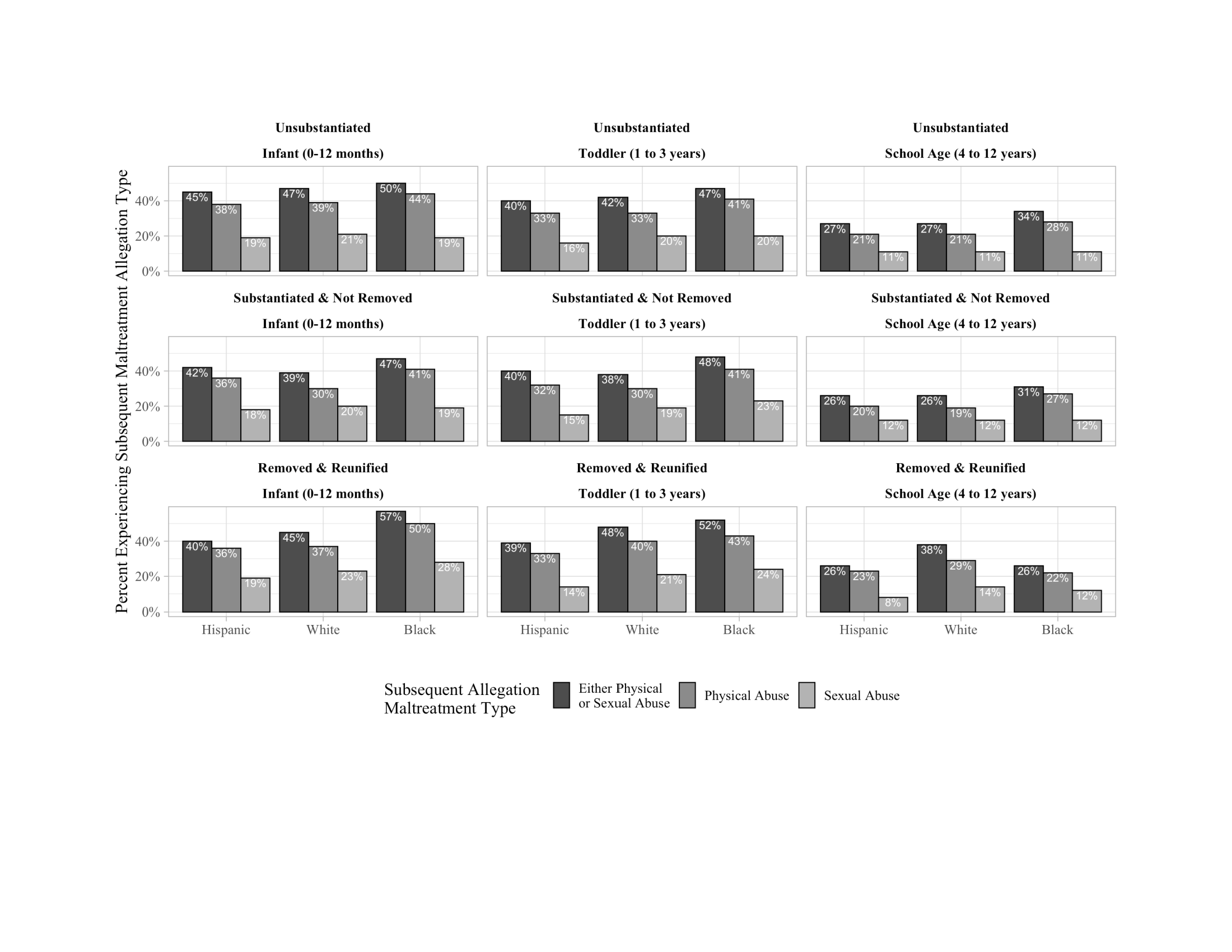

Supplement: S2 Fig — (TIF) [file pone.0283534.s002.tif]
